# Antimicrobial Activity of Ceftazidime-Avibactam and Comparators against Pathogens Harboring OXA-48 and AmpC Alone or in Combination with Other β-Lactamases Collected from Phase 3 Clinical Trials and an International Surveillance Program

**DOI:** 10.1128/aac.01985-21

**Published:** 2022-03-15

**Authors:** Lynn-Yao Lin, Dmitri Debabov, William Chang, Gregory Stone, Todd Riccobene

**Affiliations:** a Non-Clinical Development & Translational Sciences, AbbVie Inc., Irvine, California, USA; b Pfizer Inc., Groton, Connecticut, USA; c U.S. Medical Affairs, Research & Development, AbbVie Inc., Madison, New Jersey, USA

**Keywords:** AmpC, ceftazidime-avibactam, OXA-48

## Abstract

*In vitro* activities of ceftazidime-avibactam (CAZ-AVI) and key comparators against AmpC-overproducing *Enterobacterales* and Pseudomonas aeruginosa isolates from four Phase 3 clinical trials and against OXA-48–producing *Enterobacterales* with multiple resistance mechanisms from the Antimicrobial Testing Leadership and Surveillance (ATLAS) program were evaluated. Susceptibility to CAZ-AVI and comparators was determined by reference broth microdilution methods. Clinical response at test of cure (TOC) was assessed in patients from Phase 3 trials with baseline OXA-48–producing *Enterobacterales* or AmpC-overproducing *Enterobacterales* and P. aeruginosa treated with CAZ-AVI or comparators. Against 77 AmpC-overproducing *Enterobacterales* isolates from Phase 3 trials, meropenem-vaborbactam (98.7% susceptible [S]), CAZ-AVI (96.1% S), and meropenem (96.1% S) had similar *in vitro* activity and were more active than ceftolozane-tazobactam (24.7% S). Clinical cure rates in patients with baseline AmpC-overproducing *Enterobacterales* were 80.7% (*n* = 21/26) and 85.0% (*n* = 17/20) for CAZ-AVI and comparators. Against 53 AmpC-overproducing P. aeruginosa isolates from Phase 3 trials, CAZ-AVI (73.6% S) was more active *in vitro* than ceftolozane-tazobactam (58.5% S) and meropenem (37.7% S). Clinical cure rates in patients with baseline AmpC-overproducing P. aeruginosa were 85.7% (*n* = 12/14) and 75.0% (*n* = 9/12) for CAZ-AVI and comparators, respectively. Of 113 OXA-48–producing isolates from the ATLAS program, 99.1% were susceptible to CAZ-AVI. Four patients with baseline OXA-48–producing Klebsiella pneumoniae isolates treated with CAZ-AVI in Phase 3 trials were clinical cures at TOC and had favorable microbiological response. CAZ-AVI was among the most active agents against AmpC-overproducing P. aeruginosa and *Enterobacterales* and had greater *in vitro* activity against OXA-48–producing *Enterobacterales* than meropenem-vaborbactam, meropenem, ceftolozane-tazobactam, and other comparators.

## INTRODUCTION

*Enterobacterales* and Pseudomonas aeruginosa are the most common Gram-negative organisms causing serious bacterial infections with high morbidity and mortality. When these organisms produce either AmpC or OXA-48 β-lactamases, in combination with extended-spectrum β-lactamases (ESBLs), they are often resistant to most β-lactam antibiotics, resulting in very few treatment options and higher mortality (1, 2).

OXA-48 is a unique carbapenemase with low-level hydrolytic activity toward cephalosporins. Pathogens harboring *bla*_OXA-48_ usually carry other β-lactamases with high rates of ESBL coproduction that frequently result in resistance to cephalosporins as well as carbapenems (3, 4). OXA-48–producing isolates, predominantly Klebsiella pneumoniae, are increasingly prevalent in many parts of the world and are dominant in certain regions, such as North Africa, the Middle East, and Europe (1, 4, 5). A major contributor of the dissemination is the acquisition of plasmid-mediated *bla*_OXA-48_ genes. OXA-48 is inhibited by avibactam, but not inhibited by traditional β-lactamase inhibitors and other non–β-lactam-based inhibitors. Ceftazidime is not significantly hydrolyzed by OXA-48 but potentially can lose activity in the presence of ESBLs (1, 5).

AmpC β-lactamases, on the other hand, are more complex owing to inducible expression in variable levels of the chromosomal encoded *bla*_ampC_ gene (cAmpC), as well as the constitutively expressed plasmid-encoded *bla*_ampC_ genes (pAmpC) (6). A stable derepression of *bla*_ampC_ expression often results in the upregulation of expression of the AmpC β-lactamases, which in turn decreases the susceptibility of cephalosporins in pathogens that harbor the inducible system (6). Strains producing cAmpC in an inducible manner usually appear susceptible in *in vitro* assays to third-generation cephalosporins, which are weak inducers but can constitutively produce the enzymes that cause resistance to these drugs, resulting in treatment failures (2, 6). Pathogens coproducing AmpC and ESBLs may exhibit multidrug-resistant phenotypes, limiting treatment options owing to coexpression of multiple resistance determinants. AmpCs are usually resistant to β-lactam–based β-lactamase inhibitors but are well inhibited by non–β-lactam-based inhibitors, such as avibactam (6).

Ceftazidime-avibactam (CAZ-AVI) is a combination of the β-lactamase inhibitor avibactam and the broad-spectrum cephalosporin ceftazidime. Avibactam is a non–β-lactam β-lactamase inhibitor that binds reversibly to β-lactamases, efficiently inactivating β-lactamase enzymes and preventing the hydrolysis of β-lactam compounds, such as ceftazidime (7). CAZ-AVI has been used to successfully treat Gram-negative pathogens that coproduce ESBLs, OXA-48 or AmpC, and in some extreme cases of OXA-48/AmpC/ESBL–coproducing pathogens (4, 8, 9).

This study evaluated *in vitro* activities of CAZ-AVI and key comparators against AmpC-overproducing *Enterobacterales* and P. aeruginosa isolates collected from Phase 3 clinical trials, RECLAIM (10), RECAPTURE (11), REPROVE (12), and REPRISE (13) (Table 1) and OXA-48–producing *Enterobacterales* from surveillance studies. RECLAIM, RECAPTURE, and REPROVE were double-blind, noninferiority studies that evaluated the efficacy, safety, and tolerability of CAZ-AVI in the treatment of hospitalized adults with complicated intra-abdominal infections (cIAI), complicated urinary tract infections (cUTI), or hospital-acquired pneumonia/ventilator-associated pneumonia (HAP/VAP) caused by Gram-negative pathogens. REPRISE was an open-label clinical study that evaluated the efficacy of CAZ-AVI compared with best available therapy (primarily carbapenems) in patients with either cIAI or cUTI caused by ceftazidime-resistant Gram-negative pathogens. OXA-48–producing *Enterobacterales* with multiple resistance mechanisms were also collected from a CAZ-AVI global surveillance program (ATLAS: Antimicrobial Testing Leadership and Surveillance), and susceptibility of these isolates to CAZ-AVI and comparator antibiotics was tested as part of this study. Finally, clinical response at test of cure (TOC) was also assessed for patients from the Phase 3 studies that had AmpC-overproducing or OXA-48–producing *Enterobacterales* and P. aeruginosa at baseline.

**TABLE 1:** Phase 3 clinical trials^*a*^

Study title (clinicaltrials.gov registration #)	Study design	Patients	Comparator
RECLAIM 1 and 2 (NCT01499290 and NCT01500239) (10)	Prospective, randomized, double-blind, double-dummy	Adults 18–90 yrs of age with cIAI requiring surgical intervention	Meropenem
RECAPTURE 1 and 2 (NCT01595438 and NCT01599806) (11)	Two identical, prospective, randomized, double-blind, double-dummy studies	Adults 18–90 yrs of age with cUTI/pyelonephritis	Doripenem
REPROVE (NCT01808092) (12)	Prospective, randomized, double-blind, double-dummy	Adults 18–90 yrs of age with NP/VAP	Meropenem
REPRISE (NCT01644643) (13)	Prospective, randomized, open-label	Adults 18–90 yrs of age with cIAI or cUTI/pyelonephritis caused by ceftazidime-resistant Gram-negative pathogens	BAT: determined before randomization by investigator per standard of care and local label recommendation^*b*^

a BAT, best available therapy; cIAI, complicated intra-abdominal infections; cUTI, complicated urinary tract infections; NP, nosocomial pneumonia; VAP, ventilator-associated pneumonia.

b Preferred BAT: meropenem, imipenem, doripenem, or colistin (cUTI), and meropenem, imipenem, doripenem, colistin, or tigecycline (cIAI); but any therapy, including combination treatment, was permitted.

## RESULTS

### Susceptibility of OXA-48–producing isolates from surveillance data and clinical studies.

Against 113 OXA-48–producing *Enterobacterales* collected from the 2018–2019 ATLAS global program, CAZ-AVI demonstrated the highest *in vitro* activity (minimum concentration at which 50%/90% of the isolates are inhibited [MIC_50/90_], 0.5/2 mg/L; 99.1% susceptible [S]), followed by amikacin (AMK; MIC_50/90_, 4/32 mg/L; 83.2% S), meropenem-vaborbactam (MVB) (MIC_50/90_, 2/32 mg/L; 69.9% S), and gentamicin (GEN; MIC_50/90_, 2/32 mg/L; 56.6% S). Overall, the *in vitro* activities were poor for ceftazidime (MIC_50/90_, 32/32 mg/L; 26.6% S), meropenem (MEM; MIC_50/90_, 2/32 mg/L; 17.7% S), levofloxacin (LEV; MIC_50/90_, 16/16 mg/L; 17.7% S), and ceftolozane-tazobactam (TZC; MIC_50/90_, 32/32 mg/L; 14.2% S) against OXA-48–producing *Enterobacterales* (Table 2).

**TABLE 2:** MIC_50/90_ distribution of CAZ-AVI and comparator agents tested against OXA-48–producing *Enterobacterales* isolates from the ATLAS Global Surveillance Program (cumulative % at MIC)^*a*^

Antimicrobial agents	MIC mg/L										CLSI, % S
	0.25	0.5	1	2	4	8	≥16	≥32	MIC_50_	MIC_90_	
*Enterobacterales* (*n* = 113)											
CAZ-AVI	18.6	55.8	86.7	95.6	97.3	99.1	100		0.5	2	99.1
CAZ	3.0	5.3	16.8	23.0	26.5	28.3	31.0	100	32	32	26.6
MVB	2.7	8.8	26.5	54.9	69.9	80.5	85.0	100	2	32	69.9
MEM	1.8	5.3	17.7	52.2	69.9	81.4	85.0	100	2	32	17.7
TZC	2.0	2.0	5.3	14.2	26.5	35.4	44.2	100	32	32	14.2
GEN	13.3	40.7	48.7	52.2	56.6	60.2	61.1	100	2	32	56.6
LEV	10.0	17.7	26.5	29.2	34.5	38.1	100		16	16	17.7
AMK	0	5.3	27.4	40.7	71.7	83.2	87.6	100	4	32	83.2

a AMK, amikacin; ATLAS, Antimicrobial Testing Leadership and Surveillance; CAZ, ceftazidime; CAZ-AVI, ceftazidime-avibactam; CLSI, Clinical and Laboratory Standards Institute; GEN, gentamicin; LEV, levofloxacin; MEM, meropenem; MIC_50/90_, minimum concentration at which 50%/90% of the isolates are inhibited; MVB, meropenem-vaborbactam; S, susceptible; TZC, ceftolozane-tazobactam.

Molecular characterization revealed 20 out of 113 OXA-48–producing isolates carried OXA-48 alone, and the remaining 93 isolates carried additional β-lactamases. Coharbored ESBL genes were detected in 78 OXA-48 isolates (69.0% of total isolates). The most common ESBL gene types were the *bla*_CTX-M_ and *bla*_SHV_ families (CTX-M-9, 14, 15, and 55 and SHV-12). It is important to note that CAZ-AVI (98.4% S) remained highly active against OXA-48 + ESBL + limited-spectrum β-lactamase (LBSL)-coharboring isolates. These isolates had decreased susceptibility to MVB (68.9% S), ceftazidime (18.0% S), MEM (8.2% S), and TZC (4.9% S) with no major changes in susceptibility to AMK (82% S; Table 3). Plasmid-encoded AmpC genes were found in 17 OXA-48–producing isolates (15.0% of total isolates). Again, CAZ-AVI (100% S) demonstrated high *in vitro* activity against these isolates, whereas the susceptibility was decreased for MVB (64.7% S) and was very limited in MEM and TZC (each 11.8% S; Table 3).

**TABLE 3:** *In vitro* susceptibility of CAZ-AVI and comparator agents tested against *Enterobacterales* producing OXA-48 alone or in combination with other resistance mechanisms^*a*^

Resistance mechanism	% Susceptible							
	CAZ-AVI	CAZ	MVB	MEM	TZC	GEN	LEV	AMK
OXA-48 only (*n* = 20)	100	25.0	95.0	45.0	25.0	60.0	55.0	90.0
OXA-48 + LSBL (*n* = 15)	100	20.0	46.7	13.3	20.0	73.3	26.7	80.0
OXA-48 + SHV-LSBL (*n* = 10)								
OXA-48; SHV or TEM-LSBL (*n* = 5)								
OXA-48 + ESBL + LSBL (*n* = 61)	98.4	18.0	68.9	8.2	4.9	50.8	18.0	82.0
OXA-48; CTX-M-15; SHV or TEM-LSBL (*n* = 44)								
OXA-48; CTX-M-9; SHV-12 (*n* = 11)								
OXA-48; CTX-M-9,14,15,55 (*n* = 6)								
OXA-48 + AmpC (DHA, AAC, CMY) + ESBL +LSBL (*n* = 17)	100	41.2	64.7	11.8	11.8	52.9	52.9	88.2
OXA-48, CTX-M-15; CMY-6 or DHA-1; TEM or SHV-LSBL (*n* = 9)								
OXA-48; DHA-1,21 or CMY-16,42 (*n* = 6)								
OXA-48; DHA-1; TEM or SHV-LSBL (*n* = 2)								
Overall % susceptible by OXA carbapenemase (*n* = 113)	99.1	26.6	69.9	17.7	14.2	56.6	17.7	79.6

a AMK, amikacin; CAZ, ceftazidime; CAZ-AVI, ceftazidime-avibactam; CMY, cephamycin; ESBL, extended-spectrum β-lactamase; GEN, gentamicin; LEV, levofloxacin; MEM, meropenem; MVB, meropenem-vaborbactam; LSBL, limited-spectrum β-lactamase; TZC, ceftolozane-tazobactam.

There were 4 patients with OXA-48–producing isolates at baseline in the CAZ-AVI treatment group in the following Phase 3 studies conducted from 2012 to 2016: 1 from RECLAIM (cIAI), 2 from REPRISE (cIAI, cUTI with confirmed CAZ-NS pathogen), and 1 from RECAPTURE (cUTI). All 4 patients had K. pneumoniae possessing *bla_OXA-48_* in combination with additional β-lactamases (*bla_CTX-M-15_*, *bla_TEM-1_*, *bla_SHV-1_*, or *bla_OXA-1/30_*). At baseline, the CAZ-AVI MIC for all isolates was 0.5–1 mg/L and the ceftazidime MIC was >64 mg/L. All 4 K. pneumoniae isolates were resistant to MEM (MIC 4–32 mg/L), TZC (MIC ≥64 mg/L), GEN (MIC >32 mg/L), and LEV (MIC ≥16 mg/L), and susceptible to AMK (MIC 2–4 mg/L). For MVB, two isolates were susceptible (MIC 2–4 mg/mL) and two were resistant (MIC 32–64 mg/mL).

### Susceptibility of AmpC-overproducing isolates from clinical studies.

Against 77 AmpC-overproducing *Enterobacterales* isolates collected from CAZ-AVI Phase 3 clinical studies, MVB (MIC_50/90,_ 0.06/0.5 mg/L; 98.7% S), CAZ-AVI (MIC_50/90_, 0.5/2 mg/L; 96.1% S), and MEM (MIC_50/90,_ 0.12/1 mg/L; 96.1% S) exhibited similar levels of *in vitro* activity, followed by AMK (MIC_50/90,_ 2/16 mg/L; 84.4% S), GEN (MIC_50/90,_ 1/16 mg/L; 59.7% S), LEV (MIC_50/90,_ 1/8 mg/L; 45.5% S), TZC (MIC_50/90,_ 8/8 mg/L; 24.7% S), and ceftazidime (MIC_50/90,_ 16/16 mg/L; 6.5% S), according to Clinical and Laboratory Standards Institute (CLSI) breakpoint interpretation (Table 4). Coharbored ESBL genes were detected in 52% of 77 AmpC-overproducing *Enterobacterales* isolates, including 8 C. freundii complex (10% of total) and 32 E. cloacae (42% of total). The most common ESBL gene type was *bla*_CTX-M_ (40 isolates, 78% of ESBL-producing isolates). The CTX-M-15 encoding gene was detected in 26 isolates, including 6 from C. freundii complex and 20 from E. cloacae. The CTX-M-3 gene was observed in 4 C. freundii complexes and 23 E. cloacae. A total of 28 isolates carried the gene encoding OXA-1/30. This gene was mostly observed among isolates carrying other ESBLs, mainly CTX-M-15. Plasmid-encoded AmpC gene CMY-42 was noted in one *E. coli* isolate (Tables 4 and 5).

**TABLE 4:** Susceptibility of CAZ-AVI and comparators against AmpC-overproducing *Enterobacterales* and Pseudomonas aeruginosa isolates from clinical studies^*a*^

Antimicrobial agents	mg/L														CLSI, % S
	0.03	0.06	0.12	0.25	0.5	1	2	4	8	≥16	≥32	≥64	MIC_50_	MIC_90_	
*Enterobacterales* (*n* = 77)															
CAZ-AVI		5.2	13.0	29.9	57.1	79.2	92.2	94.8	96.1	100			0.5	2	96.1
CAZ		1.3	1.3	1.3	1.3	1.3	2.6	6.5	9.1	100			16	16	6.5
MVB	39.0	70.1	83.1	89.6	94.8	96.1	96.1	98.7	98.7	100			0.06	0.5	98.7
MEM	7.8	48.1	72.7	81.8	88.3	96.1	96.1	100	100				0.12	1	96.1
TZC			1.3	2.6	13.0	18.2	24.7	36.4	100				8	8	24.7
GEN				9.1	45.5	53.2	58.4	59.7	62.3	100			1	16	59.7
LEV		26.0	35.1	40.3	45.5	55.8	58.4	64.9	100				1	8	45.5
AMK	2.6	2.6	2.6	2.6	2.6	31.2	54.5	67.5	84.4	100			2	16	84.4
P. aeruginosa (*n* = 53)															
CAZ-AVI		1.9	1.9	1.9	1.9	5.7	13.2	62.3	73.6	100			4	16	73.6
CAZ		1.9	1.9	1.9	1.9	1.9	1.9	1.9	3.8	17.0	100		32	32	3.8
MVB		1.9	7.6	9.4	18.9	28.3	37.7	49.1	64.2	83.0	100		8	32	64.2^*b*^
MEM		5.7	9.4	9.4	17.0	24.5	37.7	49.1	64.2	100			8	16	37.7
TZC		1.9	1.9	1.9	1.9	15.1	43.4	58.5	77.4	100			4	16	58.5
GEN		5.7	5.7	5.7	5.7	17.0	32.1	47.2	54.7	100			8	16	47.2
LEV		13.2	13.2	13.2	13.2	17.0	17.0	17.0	22.6	100			16	16	17.0
AMK						3.8	13.2	32.1	50.9	71.7	77.4	100	8	64	71.7

a AMK, amikacin; CAZ, ceftazidime; CAZ-AVI, ceftazidime-avibactam; CLSI, Clinical and Laboratory Standards Institute; EUCAST, European Committee on Antimicrobial Susceptibility Testing; GEN, gentamicin; LEV, levofloxacin; MEM, meropenem; MIC_50/90_, minimum concentration at which 50%/90% of the isolates are inhibited; MVB, meropenem-vaborbactam; S, susceptible; TZC, ceftolozane-tazobactam.

b EUCAST breakpoint was applied.

**TABLE 5:** *In vitro* susceptibility of CAZ-AVI and comparator agents tested against *Enterobacterales* producing AmpC alone or in combination with other resistance mechanisms^*a*^

Resistance mechanism	% Susceptible							
	CAZ-AVI	CAZ	MVB	MEM	TZC	GEN	LEV	AMK
*Enterobacterales* (*n* = 77)								
Chrom. ampC overexpression only (*n* = 37)	94.4	8.3	100	97.2	28.0	94.4	89.0	100
Chrom. ampC overexpression + ESBL + other beta-lactamase (*n* = 40)	95.0	12.5	97.5	92.5	25.0	32.5	12.5	67.5
Chrom. ampC overexpression + OXA-1/30 or SHV-12 or TEM-1 (*n* = 8)								
Chrom. ampC overexpression + CTX-M-15-like + TEM-1 or OXA-1/30 or PER or NDM (*n* = 11)								
Chrom. ampC overexpression + CTX-M-15-like + OXA-1/30 + TEM-1 or DHA (*n* = 13)								
Chrom. ampC overexpression + CTX-M-15-like + CTX-M-3-like + OXA-1/30 + TEM-1 (*n* = 3)								
Chrom. ampC overexpression + CTX-M-3-like + SHV-12 + TEM-1 (*n* = 5)								
P. aeruginosa (*n* = 53)								
Chrom. ampC overexpression only (*n* = 39)	81.1	1.9	30.2	28.3	45.3	37.7	15.1	79.2
Chrom. ampC overexpression + OXA-2, or OXA-10, or OXA-14, or OXA-17, PER (*n* = 14)	71.4	1.9	64.3	35.7	50.0	35.7	1.9	64.3

a AMK, amikacin; CAZ, ceftazidime; CAZ-AVI, ceftazidime-avibactam; CMY, cephamycin; ESBL, extended-spectrum β-lactamase; GEN, gentamicin; LEV, levofloxacin; MEM, meropenem; MVB, meropenem-vaborbactam; LSBL, limited-spectrum β-lactamase; TZC, ceftolozane-tazobactam.

CAZ-AVI (MIC_50/90,_ 4/16 mg/L; 73.6% S) was among the most active agents *in vitro*, when tested against 53 AmpC-overproducing P. aeruginosa isolates collected from Phase 3 clinical studies, followed by amikacin (AMK; MIC_50/90,_ 8/64 mg/L; 71.7% S), meropenem-vaborbactam (MVB) (MIC_50/90,_ 8/32 mg/L; 64.2% S), TZC (MIC_50/90,_ 4/16 mg/L; 58.5% S), gentamicin (GEN; MIC_50/90,_ 8/16 mg/L; 47.2% S), meropenem (MEM; MIC_50/90,_ 8/16 mg/L; 37.7% S), levofloxacin (LEV; MIC_50/90,_ 16/16 mg/L; 17.0% S), and ceftazidime (MIC_50/90,_ 32/>32 mg/L; 3.8% S), when CLSI and European Committee on Antimicrobial Susceptibility Testing (EUCAST) breakpoint criteria were applied (Table 4). Coharbored OXA variants OXA-2, OXA-10, OXA-14, or OXA-17 were observed (14 isolates, 26.4% of total isolates).

Isolates included in the *in vitro* susceptibility evaluation were collected at various time points (including baseline, TOC, and end of treatment [EOT]) in the CAZ-AVI and control groups of all 4 clinical trials. The clinical outcome evaluation carried out in this study included only those patients with the isolates present at baseline. A total of 40 patients in the CAZ-AVI group, including 26 patients with baseline AmpC-overproducing *Enterobacterales*, and 14 patients with baseline AmpC-overproducing P. aeruginosa, were pooled together from the Phase 3 clinical studies: RECLAIM, RECAPTURE, REPROVE, and REPRISE. Clinical cures in patients with baseline AmpC-overproducing P. aeruginosa were 85.7% (*n* = 12/14) in the CAZ-AVI group versus 75.0% (*n* = 9/12) in the control groups. Clinical cures in patients with baseline AmpC-overproducing *Enterobacterales* were 80.7% (*n* = 21/26) in the CAZ-AVI group versus 85.0% (*n* = 17/20) in the carbapenem control groups.

## DISCUSSION

The novel β-lactam–β-lactamase inhibitor combinations (CAZ-AVI, ceftolozane-tazobactam, and meropenem-vaborbactam) are a significant advance in the therapeutic armamentarium against multidrug-resistant Gram-negative pathogens. The CAZ-AVI combination has shown potent activity against carbapenemase-producing *Enterobacterales* and P. aeruginosa owing to inhibitory activity of avibactam toward carbapenemases, including OXA-48 and KPC (2, 6). Moreover, avibactam also inhibits ESBLs and Class C cephalosporinases, offering a viable treatment option for infections caused by pathogens carrying OXA-48 or AmpC alone or in combination with ESBLs. Although the β-lactam–β-lactamase inhibitor combinations are highly effective against large collections of clinical isolates, each has a unique susceptibility profile (14). While tazobactam does not inhibit *bla*_OXA-48_, activity of ceftolozane-tazobactam may be expected against isolates with OXA-48–like enzymes, which are poorly active against ceftolozane and other oxyimino-cephalosporins. However, a study showed that ceftolozane-tazobactam displayed limited *in vitro* activity against *Enterobacterales* isolates harboring OXA-48. In a study of 353 OXA-48–producing isolates, despite the presence of tazobactam, which should inhibit ESBLs, ceftolozane-tazobactam MICs closely tracked those of unprotected ceftazidime: 81.9% of ceftazidime-susceptible/intermediate isolates (ceftazidime MIC ≤4 mg/L, EUCAST criteria) were susceptible to ceftolozane-tazobactam, with only 8.1% of ceftazidime-resistant isolates (ceftazidime MIC > 4 mg/L, EUCAST criteria) reported as susceptible to ceftolozane-tazobactam (15). The explanations include the possibility of OXA-48 overwhelming tazobactam, rendering it unable to protect ceftolozane against the AmpC or ESBL enzymes also present in these isolates, or the carriage of additional porin mutations.

Vaborbactam, a cyclic boronic acid BLI, exhibited no activity against isolates harboring *bla*_OXA-48_ when combined with meropenem (5). Previously published *in vitro* susceptibility data of MVB showed that the combination had no or limited activity against *bla*_OXA-48_ carrying *Enterobacterales* (5, 16). In this study, MVB exhibited moderate to high activity against these isolates, significantly higher than that of MEM alone with *bla*OXA-48 alone or in combination with other β-lactamases. However, the MIC_50_ and MIC_90_ of MVB were identical to those of MEM (2/32 mg/L), which is consistent with observations that vaborbactam does not inhibit *bla*_OXA-48_. The difference in susceptibility can be attributed to a higher breakpoint set for MVB (susceptible ≤4 mg/L) than that for MEM alone (susceptible ≤1 mg/L). An efficacy study of human-simulated exposure of MVB and MEM in the neutropenic murine thigh infection model revealed that the activity of both drugs against *bla*_OXA-48_ carrying *Enterobacterales* was poor despite more than a third of isolates falling within the susceptible range per EUCAST and CLSI MIC interpretation criteria (8). Therefore, caution needs to be taken when interpreting *in vitro* susceptibility data for *bla*_OXA-48_ carrying *Enterobacterales* to ensure successful clinical outcomes. In addition, vaborbactam is a potent inhibitor of Class A β-lactamases (17); however, for *Enterobacterales* carrying *bla*_OXA-48_ in combination with CTX-M, SHV, TEM, etc., this study showed that the susceptibility of MVB was dramatically lower than that of isolates carrying *bla*_OXA-48_ alone. *Enterobacterales* showed a relatively high susceptibility (80%) to AMK. The activity of AMK against isolates carrying *bla*_OXA-48_ in various combinations with ESBLs and other β-lactamases decreased by approximately 2–10%. As the presence of carbapenems or β-lactamases is not expected to impact aminoglycoside susceptibility, aminoglycoside-modifying genes, 16S rRNA methylases, and other potential mechanisms remain to be investigated. This *in vitro* susceptibility study demonstrated that CAZ-AVI was the most active agent *in vitro* against OXA-48–producing *Enterobacterales* carrying multiple β-lactamases compared with other antibiotics, including β-lactams, β-lactam–β-lactamase inhibitor combinations, and aminoglycosides.

The clinical trial data review demonstrated successful clinical outcomes for patients treated with CAZ-AVI who had infections caused by OXA-48–producing organisms, although the number of patients was small. In another observational study evaluating 57 patients receiving CAZ-AVI treatment for infections caused by OXA-48–producing *Enterobacterales*, CAZ-AVI showed promising results, even in monotherapy, for the treatment of patients with severe infections due to OXA-48–producing *Enterobacterales* and limited therapeutic options (18).

AmpC is known to be inhibited by non–β-lactam-based inhibitors, such as avibactam, although there may be some variability in susceptibility to inhibitors (2, 6). In this study, CAZ-AVI demonstrated strong *in vitro* activity against *Enterobacterales* and P. aeruginosa isolates overproducing AmpC β-lactamase along with ESBLs, whereas for ceftazidime, like other cephalosporins, the activity is compromised, resulting in limited *in vitro* activities against the same isolates. Moreover, ceftolozane-tazobactam is much less potent against the *Enterobacterales* strains overproducing AmpC β-lactamase and ESBLs owing to the weak inhibitory activity of tazobactam against inducible and constitutively expressed AmpC enzymes (18). Nevertheless, against P. aeruginosa–overproducing AmpC β-lactamase and ESBLs, the *in vitro* susceptibility to ceftolozane-tazobactam was improved.

In CAZ-AVI regulatory clinical trials evaluating the efficacy of CAZ-AVI and carbapenem comparators, the clinical success rates in patients with baseline AmpC-overproducing P. aeruginosa were 86% (*n* = 12/14) in the CAZ-AVI group versus 75% (*n* = 9/12) in the carbapenem control groups. Clinical success rates in patients with baseline AmpC-overproducing *Enterobacterales* were 81% (*n* = 21/26) in the CAZ-AVI group versus 85% (*n* = 17/20) in carbapenem groups. Similar results were observed in a systematic review and meta-analysis of randomized controlled trials comparing the clinical efficacy of CAZ-AVI and carbapenems for the treatment of ESBL/AmpC-producing *Enterobacterales*; the clinical response for AmpC producers in CAZ-AVI and carbapenem arms was 80% (*n* = 32/40) and 88% (*n* = 37/42), respectively (2). These results showed evidence of the clinical efficacy of ceftazidime-avibactam as a potential alternative to carbapenems in patients with AmpC-overproducing or OXA-48–producing Enterobacteriaceae and P. aeruginosa. In a retrospective multicenter study evaluating clinical success in patients hospitalized in 13 Italian hospitals who received ≥72 h of CAZ-AVI, a 90% clinical cure rate was observed in all 41 assessed patients at the end of CAZ-AVI treatment despite the study population having a higher prevalence of infections caused by MDR, XDR, and PDR, including OXA-48, AmpC, ESBL, and KPC pathogens (19).

In this descriptive analysis, the number of isolates at baseline overproducing AmpC β-lactamase or OXA-48 carbapenemase was small, limiting interpretation of the clinical outcome evaluation. Additional limitations are that the data were pooled from four clinical trials that had differences in design, limiting direct comparison. As a result, the analyses carried out were exploratory, and the results should be interpreted with caution.

In conclusion, our study showed that CAZ-AVI was among the most active agents against AmpC-overproducing P. aeruginosa and *Enterobacterales* and had greater *in vitro* activity against OXA-48–producing *Enterobacterales* than comparators.

## MATERIALS AND METHODS

### Bacterial isolates.

Nonduplicate clinical isolates of AmpC-overproducing *Enterobacterales* (*n* = 77) and P. aeruginosa (*n* = 53) were collected from 4 CAZ-AVI clinical trials: RECLAIM (cIAI; NCT01499290/NCT01500239), REPRISE (cIAI/cUTI; NCT01644643), RECAPTURE (cUTI; NCT01595438/NCT01599806), and REPROVE (HAP/VAP; NCT01808092). AmpC-overproducing *Enterobacterales* included Enterobacter cloacae (*n* = 49), Citrobacter freundii complex (*n* = 14), Enterobacter aerogenes (*n* = 8), Escherichia coli (*n* = 5), and Serratia marcescens (*n* = 1). The isolates included in the *in vitro* susceptibility evaluation were collected at various time points, including baseline, TOC, and EOT in the CAZ-AVI group and in the comparator study treatment groups.

Four OXA-48–producing K. pneumoniae isolates were collected from CAZ-AVI clinical trials (1 from RECLAIM, 2 from REPRISE, and 1 from RECAPTURE). Nonduplicate clinical isolates of OXA-48–producing *Enterobacterales* (*n* = 113) were collected from the 2018–2019 Antimicrobial Testing Leadership and Surveillance (ATLAS) global program by medical centers in 25 countries. The OXA-48–producing isolates included K. pneumoniae (*n* = 65), Klebsiella oxytoca (*n* = 6), Klebsiella aerogenes (*n* = 5), E. cloacae (*n* = 17), Enterobacter kobei (*n* = 2), E. coli (*n* = 11), and others (*n* = 4).

### Resistant subsets.

β-Lactamase gene screening in AmpC-overproducing clinical trial isolates was conducted using either microarrays (Check-Points, Wageningen, Netherlands) and PCR or a combination of both as described previously (20–23). The presence and expression levels of AmpC were determined using quantitative PCR, and the threshold of upregulation was 5-fold above a reference value as described previously (22). The screening was conducted at central reference laboratories (JMI, North Liberty, IA, USA).

OXA-48–producing *Enterobacterales* isolates were screened for genes encoding carbapenemases (KPC, OXA-48–like, NDM, IMP, VIM) as well as the presence of coharbored β-lactamase genes encoding TEM, SHV, CTX-M-1 group, CTX-M-2 group, CTX-M-8 group, CTX-M-9 group, CTX-M-25 group, ACC, ACT, CMY, and DHA using a combination of microarray and multiplex PCR assays, followed by amplification and sequencing of the full-length genes. The screening was conducted at the central reference laboratory (IHMA, Schaumburg, IL, USA).

### Susceptibility testing.

*In vitro* susceptibility testing was performed by the broth microdilution (BMD) method, using a custom-made panel manufactured by ThermoFisher Inc. (Waltham, MA, USA) consisting of CAZ-AVI, ceftazidime, meropenem (MEM), meropenem-vaborbactam (MVB), ceftolozane-tazobactam (TZC), gentamicin (GEN), levofloxacin (LEV), and amikacin (AMK).

Antimicrobial susceptibility was conducted according to Clinical and Laboratory Standards Institute (CLSI) procedures (document M07) (24). Avibactam was provided by Allergan (Irvine, CA, USA; prior to its acquisition by AbbVie) and combined with ceftazidime (avibactam at fixed concentration of 4 μg/mL) for susceptibility testing. CLSI susceptibility interpretive criteria were used to determine susceptibility/resistance rates. European Committee on Antimicrobial Susceptibility Testing (EUCAST) susceptibility interpretive criteria were used for MVB against P. aeruginosa (25).

### Clinical outcome evaluation.

In this study, the clinical response at TOC was reviewed in patients with baseline AmpC- overproducing *Enterobacterales* and baseline AmpC-overproducing P. aeruginosa, as well as baseline OXA-48–producing *Enterobacterales*, treated with CAZ-AVI or carbapenem comparators. Because the primary endpoint and analysis populations for the Phase 3 studies differed depending on the study and regulatory authority (U.S. Food and Drug Administration or European Medicines Agency), the clinical response (cure, failure, or indeterminate) at TOC visit in the microbiologically modified intention-to-treat (mMITT) population was selected for comparisons. Briefly, clinical cure was defined as complete resolution or substantial improvement of signs and symptoms of the infection, such that no further antibacterial therapy (other than those allowed per protocol) was necessary. TOC was assessed 21 to 25 days after randomization (REPRISE, RECAPTURE, and REPROVE) or 28 to 35 days after randomization (RECLAIM).
